# Significance of spinal cord perfusion pressure following spinal cord injury: A systematic scoping review

**DOI:** 10.1016/j.jcot.2022.102024

**Published:** 2022-09-11

**Authors:** Cameron M. Gee, Brian K. Kwon

**Affiliations:** a Department of Orthopaedics, Faculty of Medicine, University of British Columbia, Canada; b International Collaboration on Repair Discoveries, Faculty of Medicine, University of British Columbia, Canada

**Keywords:** Hemodynamic management, Intrathecal pressure, Intraspinal pressure, Spinal cord perfusion pressure, Tetraplegia, Paraplegia

## Abstract

This scoping review systematically reviewed relevant research to summarize the literature addressing the significance of monitoring spinal cord perfusion pressure (SCPP) in acute traumatic spinal cord injury (SCI). The objectives of the review were to (1) examine the nature of research in the field of SCPP monitoring in SCI, (2) summarize the key research findings in the field, and (3) identify research gaps in the existing literature and future research priorities.

Primary literature searches were conducted using databases (Medline and Embase) and expanded searches were conducted by reviewing the references of eligible articles and searches of Scopus, Web of Science core collection, Google Scholar, and conference abstracts. Relevant data were extracted from the studies and synthesis of findings was guided by the identification of patterns across studies to identify key themes and research gaps within the literature.

Following primary and expanded searches, a total of 883 articles were screened. Seventy-three articles met the review inclusion criteria, including 34 original research articles. Other articles were categorized as conference abstracts, literature reviews, systematic reviews, letters to the editor, perspective articles, and editorials. Key themes relevant to the research question that emerged from the review included the relationship between SCPP and neurological recovery, the safety of monitoring pressures within the intrathecal space, and methods of intervention to enhance SCPP in the setting of acute traumatic SCI.

Original research that aims to enhance SCPP by targeting increases in mean arterial pressure or reducing pressure in the intrathecal space is reviewed. Further discussion regarding where pressure within the intrathecal space should be measured is provided. Finally, we highlight research gaps in the literature such as determining the feasibility of invasive monitoring at smaller centers, the need for a better understanding of cerebrospinal fluid physiology following SCI, and novel pharmacological interventions to enhance SCPP in the setting of acute traumatic SCI. Ultimately, despite a growing body of literature on the significance of SCPP monitoring following SCI, there are still a number of important knowledge gaps that will require further investigation.

## 1 Introduction

Following traumatic spinal cord injury (SCI) there are currently few available treatment options to potentially improve neurologic outcome.^1^ One widely implemented option is aggressive hemodynamic management in the first seven days following SCI to mitigate sequalae associated with SCI-induced systemic hypotension and spinal cord ischemia (i.e. secondary injury^2^). As such, current guidelines for the hemodynamic management of acute SCI recommend the maintenance of mean arterial blood pressure (MAP) between 85 and 90 mmHg for the first seven days following injury.^3^^,^^4^ However, these guidelines are based largely upon retrospective studies or uncontrolled case reports and series^5^ and questions remain about not only the effectiveness but the feasibility of adhering to these guidelines in practice.^6^^,^^7^

Recent evidence has indicated that spinal cord perfusion pressure (SCPP) is more closely related to neurologic recovery than is MAP.^8^ SCPP represent the net pressure gradient that drives oxygen delivery to the injured cord and is calculated as the difference between MAP and the pressure within the intrathecal space.^9^ In the setting of acute traumatic SCI, MAP is typically measured by intra-arterial catheter (e.g. radial artery line) or an external blood pressure cuff. Pressure within the intrathecal space can be measured either by surgically inserting a pressure probe at the site of the injury during spinal decompression/fusion, or by percutaneous insertion of a catheter into the lumbar cistern distal to the injury site. The former has been termed the ‘intraspinal pressure’ (ISP) as the pressure potentially reflects the pressure inside the spinal cord when the injured cord swells against the dura and compresses against the pressure probe, while the latter is referred to as the ‘intrathecal pressure’ (ITP) or ‘cerebrospinal fluid (CSF) pressure’. Because inserting pressure monitoring probes into the intrathecal space (either at the injury site or the lumbar cistern) is not typically performed in acute traumatic SCI patients, the hemodynamic management of SCI has traditionally focused solely on the MAP.

Monitoring of SCPP following traumatic SCI may be considered analogous to the monitoring of cerebral perfusion pressure (equal to the difference between MAP and intracranial pressure) in the setting of traumatic brain injury. Notably, recommendations for the hemodynamic management of traumatic brain injury provide level IIB recommendations regarding cerebral perfusion pressure thresholds and level III regarding blood pressure thresholds.^10^ Similarly, SCPP is routinely monitored during thoraco-abdominal aortic aneurysm repair where the spinal cord's blood supply is inherently vulnerable.^11^ In this setting, a catheter is inserted percutaneously into the lumbar cistern and used to both monitor ITP and to drain CSF to lower ITP (and thus raise SCPP).^12^ Considering SCPP in the setting of acute traumatic SCI may provide an elegant solution to mitigate secondary injury to the cord. Despite what appears to be sound rationale for monitoring SCPP in SCI, few original investigations have examined its significance or the most appropriate method of calculating SCPP (i.e. ISP or ITP).

In undertaking this review we acknowledge the systematic reviews of others on related topics.^5^^,^^13^, ^14^, ^15^ Given the limited number of studies in the field and the heterogeneity of article types and outcome measures we deemed a scoping review to be appropriate. The advantage of a scoping review being that it allows for a systematic review that addresses the broader topic of monitoring SCPP in acute SCI wherein many different study designs and outcome measures may be applicable. In doing so, we do not specifically seek to address a specific research question but rather identify all relevant literature in an iterative fashion.^16^ As such, the objectives of this review are to (1) examine the nature of research in the field of SCPP monitoring in SCI, (2) summarize the key research findings in the field, and (3) identify research gaps in the existing literature and future research priorities.

## 2 Methods

This scoping review followed the five stage scoping review methodological framework of Arksey and O'Malley.^16^ In addition we followed guidance of the Joanna Briggs Institute Manual for Evidence Synthesis^17^ and the checklist of Preferred Reporting Items for Systematic reviews and Meta-Analyses (PRISMA) extension for Scoping Reviews.^18^ The protocol for this review was published on Open Science Framework on 2022/04/25 and can be found at *https://osf.io/ymq8d/**.*

Stage 1: identify the research question

The primary research question for this scoping review was: “what is known of the significance of monitoring SCPP following acute traumatic SCI?”

Stage 2: identify relevant studies

**Fig. 1:**
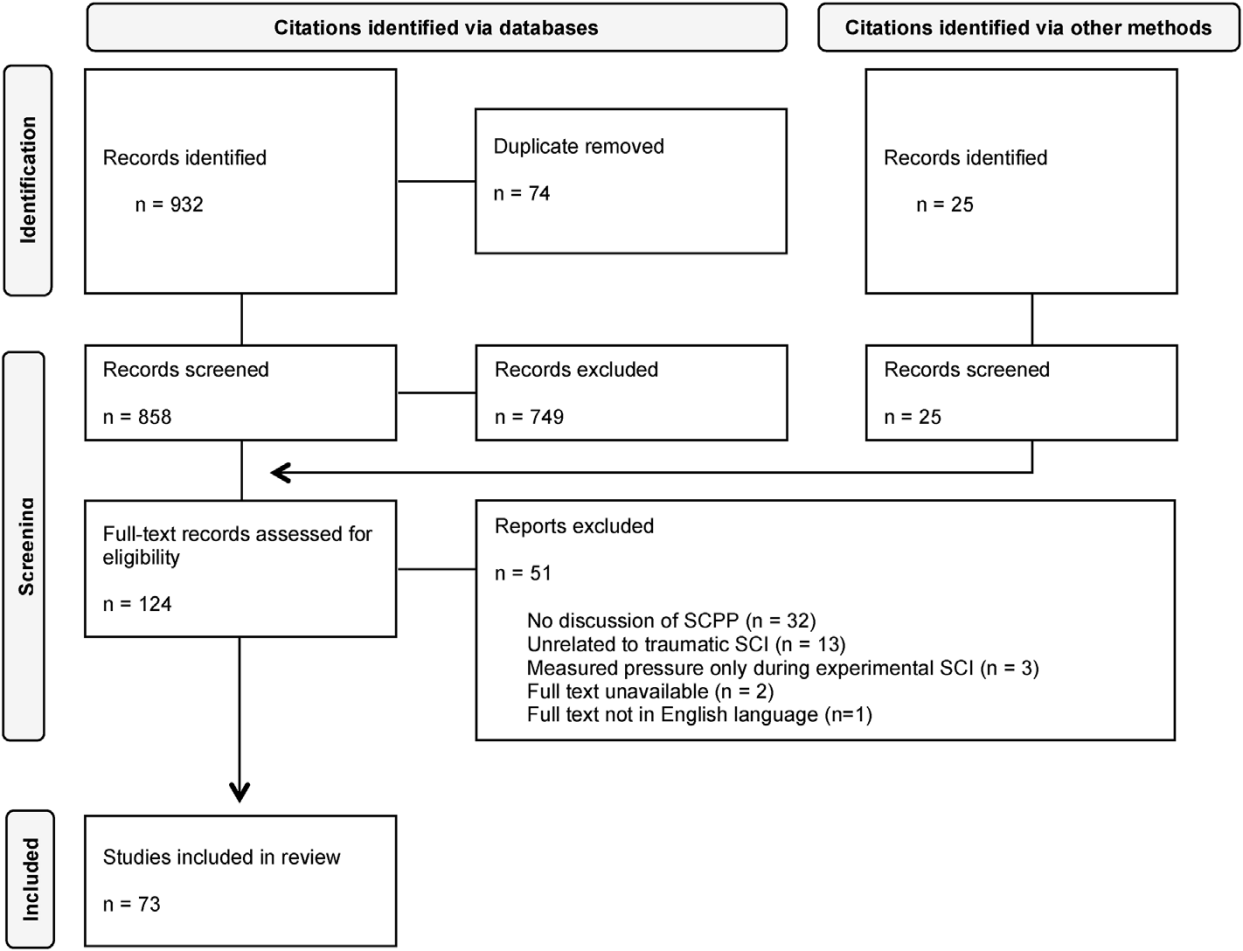
PRISMA flowchart of systematic literature search for research articles relevant to the monitoring of spinal cord perfusion pressure following acute traumatic spinal cord injury.

The search strategy was developed in consultation with a Reference Librarian at the University of British Columbia's Biomedical Branch Library. The first author (CMG) conducted searches within the databases Medline (Ovid) and Embase (Ovid) for articles published online up to and including April 27, 2022. Search terms were ‘spinal cord injur∗’, ‘tetraplegi∗’, ‘quadriplegi∗’, OR ‘paraplegi∗’ AND ‘spinal cord perfusion pressure’, ‘intraspinal pressure’, ‘intrathecal pressure’, OR ‘cerebrospinal fluid pressure’. All articles were uploaded to the database software platform Covidence where duplicate citations were identified and removed, and remaining abstracts were screened for eligibility. Expanded searches were conducted by reviewing citations and references from eligible articles retrieved in the primary search and additional searches of Scopus, Web of Science core collection, Google Scholar, and conference abstracts (see Fig. 1).

Stage 3: study selection

Studies were included if they measured, or were relevant to the concept of monitoring, SCPP in the context of traumatic SCI. Importantly, studies were not excluded based on outcome measure or study design. As such, both clinical and pre-clinical studies, interventional and observational, reviews, conference abstracts, and/or commentaries were eligible for inclusion. Articles were excluded if they were specific only to non-traumatic SCI (e.g. in the setting of thoraco-abdominal aortic aneurysm), and the search was limited to only those published in the English language. All selected studies were agreed upon by authors before charting of the data. A PRISMA flowchart^19^ outlining the search strategy process can be seen in Fig. 1.

Stage 4: charting the data

Data was charted using a pre-determined data abstraction template adapted from the Cochrane Handbook for Systematic Reviews of Interventions.^20^ Authors discussed, and agreed upon, the tool before abstracting data to ensure clarity and consistency. The first author charted the following information from each article: author(s), title, year of publication, country, type of article, purpose of the article, and key findings as they related to the research question. Where appropriate the following data was charted: setting (e.g. clinical, pre-clinical), sample size, patient age, sex, injury level, injury severity, how pressure within the intrathecal space was assessed (i.e. ISP at the injury site or ITP in the lumbar cistern), and outcome measures. The senior author checked the abstractions to ensure that all relevant information was obtained from the included articles and any discrepancies between authors were resolved through discussion. The authors were not blinded during the data abstraction and charting process.

Stage 5: collating, summarizing, and reporting the results

Results were collated and summarized consistent with the recommendations from the Joanna Briggs Institute.^17^ Given the heterogeneity of outcome measures reported, key findings were summarized using narrative synthesis (rather than data synthesis). Synthesis of findings was guided by the identification of patterns across studies. Risk of bias assessments was not performed as they are not congruent with the objectives of scoping reviews.^16^

## 3 Results

### 3.1 Articles retrieved

The database search yielded a total of 858 unique citations. Following screening of titles and abstracts for inclusion/exclusion criteria for eligibility and an expanded search, 124 full-texts were retrieved and assessed for eligibility. Following full-text screening, 73 unique citations were included in the data extraction process (see Fig. 1). Tables outlining full-text screening inclusion/exclusion and reasons for exclusion of individual articles are available on the Open Science Framework.

### 3.2 Article characteristics

The full data extraction table for all 73 included articles is provided on the Open Science Framework. These articles consisted 34 original research articles,^6^^,^^8^^,^^9^^,^^21^, ^22^, ^23^, ^24^, ^25^, ^26^, ^27^, ^28^, ^29^, ^30^, ^31^, ^32^, ^33^, ^34^, ^35^, ^36^, ^37^, ^38^, ^39^, ^40^, ^41^, ^42^, ^43^, ^44^, ^45^, ^46^, ^47^, ^48^, ^49^, ^50^, ^51^ 13 conference abstracts,^52^, ^53^, ^54^, ^55^, ^56^, ^57^, ^58^, ^59^, ^60^, ^61^, ^62^, ^63^ 13 literature reviews,^24^^,^^64^, ^65^, ^66^, ^67^, ^68^, ^69^, ^70^, ^71^, ^72^, ^73^, ^74^, ^75^ 4 systematic reviews,^5^^,^^13^, ^14^, ^15^ 4 letters to the editor,^76^, ^77^, ^78^, ^79^ 3 perspective articles,^80^, ^81^, ^82^ and 2 editorials.^83^^,^^84^ One article was published in 1979 and all other between 2008 and 2022. Specifically, articles were published in 2008 (n = 1), 2009 (n = 1), 2011 (n = 2), 2013 (n = 4), 2014 (n = 6), 2015 (n = 13), 2016 (n = 7), 2017 (n = 9), 2018 (n = 3), 2019 (n-4), 2020 (n = 8), 2021 (n = 10), and 2022 (n = 4). Primary affiliations demonstrated that research was conducted in ten countries: United Kingdom (n = 32), United States (n = 17), Canada (n = 11), Australia (n = 4), China (n = 3), Germany (n = 2), Denmark, France, Pakistan, and South Korea (all n = 1).

Sub-analysis of original research articles indicated that 24 studies had observational study designs and ten had an interventional design. Select descriptive data extracted from original research articles is presented in Table 1, Table 2.

**Table 1:** Data extraction from original articles in the clinical setting.

Citation	Sample size	*Age (years)*	*Sex (M/F)*	LOI	AIS	Pressure measure method	AEs related to monitoring	Functional recovery assessment	Key outcome measures
Kwon et al. (2009)	22	41 ± 3	15/7	17C, 5T	A- C	ITP	Headache (n = 1)	Yes	ITP, MAP, SCPP, CSF drainage, AIS
	***Key findings:*** Intrathecal catheter insertion and drainage not associated with significant AEs. CSF drainage was conservative (maximum 10 mL/h) and had minimal effect on ITP. Mean post-operative ITP was 17.4 mmHg in the group that underwent CSF drainage and 18.7 mmHg in the control group. CSF drainage was not associated with functional recovery. Suggest higher CSF drainage rate.								
Kong et al. (2013)	21	45 ± 18	20/1	10C, 11T	A- C	ITP	N/A	No	ITP, MAP, SCPP
	***Key findings:*** All patients had SCPP <60 mmHg at some point and the majority fell <50 mmHg. Mean ITP was 16.7 mmHg and SCPP 67.0 mmHg. Despite average MAP meeting target, unable to consistently maintain MAP at target for duration of the observation period.								
Werndle et al. (2014)	18	45	11/7	C5-T12	A- C	ISP	No	Yes	ISP, SCPP, waveform analysis, SCBF, MEPs, PaCO_2_, sevoflurane, mannitol, and inotrope dose, AIS
	***Key findings:*** ISP pressure was higher than that recorded below the injury site. SCPP was <60 mmHg for 16.5% of the first seven days post-SCI. Changes in PaCO_2_, sevoflurane dose, mannitol dose did not affect ISP or SCPP but inotropes increased SCPP.								
Phang et al. (2015)	1	25	1/0	T3	A	ISP & ITP	N/A	No	ISP, ITP
	***Key findings:*** ISP at injury site was >10 mmHg higher than recorded above or below the injury. After severe SCI there are three intradural compartments and monitoring below the injury site is inadequate when the cord is compressed against the dura.								
Varsos et al. (2015)	18	UNK	UNK	UNK	A- C	ISP	N/A	No	ISP, MAP, SCPP, waveform analysis
	***Key findings:*** Waveform morphology of ISP following SCI is similar to that of the intracranial pressure waveform following TBI.								
Phang et al. (2015b)	21	43 ± 3	15/6	11C, 10T	A- C	ISP	Pseudomeningocele (n = 5), CSF leak (n = 1)	No	ISP, SCPP, MRI
	***Key findings:*** Duroplasty expands intradural space and more effectively decompresses the cord resulting in lower ISP and higher SCPP.								
Czosnyka et al. (2016)	10	UNK	UNK	UNK	A- C	ISP	N/A	No	ISP, SCPP, waveform analysis
	***Key findings:*** ISP waveform similar to that of intracranial pressure in setting of TBI and similar methods of analysis may be applicable.								
Phang et al. (2016)	14	38	13/1	6C, 8T	A- C	ISP	Pseudomeningocele (n = 3), CSF leak (n = 1)	No	ISP, SCPP, injury site metabolism, dexamethasone conc.
	***Key findings:*** SCPP is major determinant of drug delivery and should be 90–100 mmHg to reduce metabolic derangement at injury site.								
Phang et al. (2016b)	42	UNK	UNK	C4-L2	A- C	ISP	Pseudomeningocele (n = 8), CSF leak (n = 3)	No	AEs, demographics, injury to surgery time, patient posture, MRI
	***Key findings:*** ISP pressure probes are accurate and safe for up to one-week post-SCI. Complications include pseudomeningocele and CSF leak. ISP was higher in laminectomized patients in the supine position and should be avoided to prevent elevated ISP.								
Altaf et al. (2017)	11	38	8/3	C5-T6	A- C	ITP	N/A	No	ITP, MAP, SCPP
	***Key findings:*** Compared to dopamine, norepinepherine was able to maintain MAP with a lower ITP (17 vs. 20 mmHg) and higher SCPP. Norepinepherine may be preferable to dopamine if vasopressor support is required to maintain MAP in acute SCI.								
Chen et al. (2017)	45	UNK	35/10	25C, 20T/L	A- C	ISP	N/A	Yes	ISP, MAP, SCPP, AIS, injury site metabolism
	***Key findings:*** SCPP deviation from optimal SCPP was associated with poorer neurological recovery. Maintaining optimal SCPP appears to enhance injury site metabolism. However, optimal SCPP could only be computed 45% of the time.								
Saadoun et al. (2017)	45	41	36/9	C2-L2	A- C	ISP	N/A	Yes	ISP, MAP, SCPP, AIS
	***Key findings:*** Strong correlation between neurological recovery and low ISP, high SCPP. SCPP >90 mmHg associated with recovery.								
Squair et al. (2017)	92	43 ± 16	72/20	55C, 28T, 9L	A- C	ITP	N/A	Yes	ITP, MAP, SCPP, AIS
	***Key findings:*** Data indicate that a target SCPP of 60–65 mmHg may be optimal and this can be achieved with good patient tolerance.								
Chen et al. (2018)	49	41 ± 2	39/10	27C, 22T	A- C	ISP	N/A	Yes	ISP, SCPP, AIS
	***Key findings:*** High ISP and low SCPP strongly correlate with reduced multi-scale entropy. Further research required to define clinical value of non-linear dynamical analysis of ISP.								
Chen et al. (2018b)	58	41 ± 2	46/12	30C, 25T, 3L	A- C	ISP	Pseudomeningocele (n = 15), CSF leak (n = 8)	Yes	ISP, MAP, SCPP, AIS
	***Key findings:*** Visibility graph analysis of ISP sensitive to pathologies such as compression, ischemia, deranged autoregulation.								
Hogg et al. (2019)	64	42	49/15	33C, 22T, 9L	A- C	ISP	N/A	No	ISP, MAP, SCPP
	***Key findings:*** Modifiable factors that may lower ISP and elevate SCPP include reducing surgical bleeding and expansion duroplasty.								
Squair et al. (2019)	92	43 ± 6	72/20	55C, 28T, 9L	A- C	ITP	N/A	Yes	ITP, MAP, SCPP, AIS
	***Key findings:*** SCPP is independently associated with neurological recovery whereas MAP is not. Maintaining SCPP above 50 mmHg is a strong predictor of improved recovery.								
Gallagher et al. (2020)	12	40 ± 18	8/4	7C, 5T/L	A- C	ISP	CSF leak (n = 3)	Yes	ISP, MAP, SCPP, spinal cord metabolism, SEPs, AIS
	***Key findings:*** Higher SCPP may be beneficial or detrimental. Suggest individualized management guided by ISP monitoring at injury site rather than applying general MAP recommendations.								
Gallagher et al. (2020b)	5	45 ± 7	3/2	T3-L1	A- C	ISP	N/A	Yes	ISP, SCPP, spinal cord metabolism and inflammation, AIS
	***Key findings:*** Spinal cord cooling did not affect ISP or SCPP, however rewarming was associated with elevated ISP, decreased SCPP, unfavourable cord metabolism, and cord inflammation. Study terminated early due to AEs unrelated to pressure monitoring.								
Hogg et al. (2020)	13	47	13/0	C3-L1	A- C	ISP & ITP	Pseudomeningocele (n = 6), CSF leak (n = 4)	No	ISP, ITP, MAP, SCPP, CSF drainage, spinal cord metabolism, AIS
	***Key findings:*** Monitoring from lumbar CSF provides limited information on injury site. ISP ∼5 mmHg higher than ITP. CSF drainage did not influence ISP in 7/12 patients and led to drop of >5 mmHg in 5/12 - likely related to degree of spinal cord swelling.								
Yue et al. (2020)	15	61 ± 17	11/4	14C, 1T	A-D	ITP	No	Yes	ITP, SCPP, AIS, length of hospital stay.
	***Key findings:*** SCPP target directed care is feasible and without complications.								
Hogg et al. (2021)	14	47	13/1	C2-L1	A- C	ISP	Pseudomeningocele (n = 7), chest sepsis (n = 5)	No	ISP, MAP, SCPP, anorectal manometry
	***Key findings:*** Higher SCPP is associated with acute improvements in anal continence. Too high of a SCPP may worsen anal continence. Highest resting anal pressures recorded when SCPP was ∼100 mmHg.								
Hogg et al. (2021b)	19	47	14/5	C4-L1	C	ISP	Pseudomeningocele (n = 4), CSF leak (n = 2), chest sepsis (n = 4)	Yes	ISP, MAP, SCPP, AIS, spinal cord metabolism
	***Key findings:*** Normalizing SCPP and ISP likely improves limb power in patients with motor incomplete SCI at admission.								
Gee et al. (2022)	16	40 ± 19	13/3	C4-T11	A, C	ITP	N/A	No	ITP, MAP, SCPP, vasopressor dose, AIS
	***Key findings:*** ITP and SCPP are highly variable. SCPP was <65 mmHg on 14% and ITP >15 mmHg on 39% of measurements.								
Hogg et al. (2022)	13	47	12/1	8C, 4T, 1L	A- C	ISP	Pseudomeningocele (n = 7), Syrinx (n = 1)	No	ISP, MAP, SCPP, urodynamics, psychosocial measures
	***Key findings: H***igh and low SCPP associated with unfavourable urodynamics. Optimizing SCPP may improve urological outcomes.								
Visagan et al. (2022)	26	43	21/5	14C, 10T, 2L	A- C	ISP	CSF leak (n = 5), wound infection (n = 1)	Yes	ISP, SCPP, spinal cord metabolism, cord tissue oxygen, CSF drainage, AIS
	***Key findings:*** Cord tissue oxygen was greater when ISP was <10 mmHg and SCPP was >90 mmHg. Patients with motor-complete SCI had lower tissue oxygen. CSF was drained to assess the effect on tissue oxygen but not ISP.								

*Abbreviations:* AE, adverse event; AIS, American Spinal Injury Association Impairment Scale; C, cervical spinal level (as it relates to LOI); CSF, cerebrospinal fluid; MAP, mean arterial pressure; MEP, motor evoked potential; MRI, magnetic responance imaging; ISP, intraspinal pressure; ITP, intrathecal pressure; L, lumbar spinal level; LOI, level of injury; PaCO_2_, partial pressure of arterial carbon dioxide; SCBF, spinal cord blood flow; SCI, spinal cord injury; SCPP, spinal cord perfusion pressure; SEP, sensory evoked potential; T, thoracic spinal level; TBI, traumatic brain injury; UNK, unknown.

**Table 2:** Data extraction from original articles in the pre-clinical setting.

Citation	Sample size	LOI	Pressure measure method	Functional recovery assessment	Key outcome measures
Griffiths et al. (1979) *J Neurosurg Spine*	12	L2	ISP	Yes	SCPP, SCBF, vascular resistance, SEPs
	***Key findings:*** Hypotension alone did not influence SCBF or SEPs. Compression combined with hypotension reduced both SCBF and evoked potentials. With compression, SCBF was maintained until a SCPP of ∼<70 mmHg. In the setting of cord compression, the cord will become ischemic once the autoregulatory limit is passed.				
Horn et al. (2008) *Neurosurg Focus*	16	T9	ITP	Yes	ITP, MAP, SCBF, MEPs, CSF drainage, motor exams
	***Key findings:*** CSF can be drained via lumbar intrathecal catheter to reduce ITP in rabbits. CSF drainage reduced the area of tissue damage at the injury site but was not associated with functional recovery.				
Batchelor et al. (2011) *J Neurotrauma*	15 (12 SCI, 3 control)	T7	ISP	Yes	ISP, MAP, core temperature, behavioural assessment, histology
	***Key findings:*** Following SCI in a rodent model, ISP rapidly rises with progressive canal occlusion and is associated with neurological deterioration. Hypothermia rapidly reduces ISP and may be useful to aid decompression prior to surgery.				
Leonard et al. (2013) J *Neurotrauma*	88	T10	ISP	Yes	ISP, histology, functional assessment, edema measurement
	***Key findings:*** Substance P mediates neurogenic inflammation and associated increased edema and ITP. Data implicate the aquaporin 4 water channel in development of edema in SCI. These may be potential therapeutic targets for future studies to limit inflammation and edema.				
Soubeyrand et al. (2013) *Eur Spine J*	27 (17 SCI, 10 control)	T10	ITP	No	ITP, MAP, SCBF
	***Key findings:*** SCI reduced SCBF and elevated ITP for up to 60 min following injury. The rodent model allows ITP and SCBF measurements following experimental SCI.				
Martirosyan et al. (2015) *Neurosurg*	15 (12 SCI, 3 control)	T5	ITP	No	ITP, MAP, CSF drainage, SCBF
	***Key findings:*** MAP elevation combined with CSF drainage increased SCBP 24%. MAP elevation or CSF drainage alone, did not.				
Leonard et al. (2015) *J Neurotrauma*	66	T10	ISP	No	ISP, histology, edema measurement
	***Key findings:*** Elevated ISP (and reduced SCPP) is associated with both increases in hemorrhage and edema at varying time points. The initial rise in ISP was associated with hemorrhage while the later increase was due to increases tissue water content.				
Khaing et al. (2017) *J Neurotrauma*	36	T7	ISP	No	ISP, MAP, SCPP
	***Key findings:*** ISP increases threefold in first 30 min following injury and remains elevated for up to seven days. In the first 24 h following SCI dural and pial linings contribute equally to the rise in ISP whereas after 72 h the dural lining is primarily responsible.				

*Abbreviations:* CSF, cerebrospinal fluid pressure; L, lumbar spinal level; LOI, level of injury; MAP, mean arterial pressure; MEP, motor evoked potential; ISP, intraspinal pressure; ITP, intrathecal pressure; SCBF, spinal cord blood flow; SCI, spinal cord injury; SCPP, spinal cord perfusion pressure; SEP, sensory evoked potential; T, thoracic spinal level.

### 3.3 Outcome measures reported

Description of outcome measures reported by each original research article are presented in Table 1, Table 2

Twenty-six original research articles were conducted in the clinical setting and eight in the pre-clinical setting. Pre-clinical studies were conducted in rabbit (n = 3)^28,32,33^, rodent (n = 3)^29,40,46^, canine,^22^ and porcine^34^ (both n = 1) models of SCI. All 34 original research articles included a measure of pressure within the intrathecal space. Twenty-two articles measured only ISP, ten measured only ITP, and two articles measured both ISP and ITP. ISP (i.e. pressure measured at the injury site) was also termed cord pressure,^22^ intracanal pressure,^46^ intraparenchymal pressure,^29^ and intrathecal pressure.^32^^,^^33^ ITP was also referred to as CSF pressure^8^^,^^40^^,^^42^ and intraspinal pressure.^45^ The only clinical studies to monitor ISP were all conducted by the same research group in the United Kingdom (n = 19) and 6/7 clinical studies to monitor ITP were conducted by a single research group in Canada.

Seventeen original research articles assessed functional recovery. All clinical trials that assessed functional recovery used the International Standards for the Neurological Classification of Spinal Cord Injury (i.e. the AIS exam).^85^ One clinical study assessed motor evoked potentials^9^ and one assessed sensory evoked potentials.^21^ Pre-clinical trials incorporated sensory evoked potentials,^22^ motor examinations,^28^ and other functional assessments specific to the pre-clinical model.^32^^,^^46^

Among all original research articles, eight studies attempted to enhance SCPP. Seven studies endeavored to do so by reducing pressure within the intrathecal space – four by CSF drainage,^27^^,^^28^^,^^31^^,^^34^ two by spinal cord cooling,^46^^,^^51^ and one by expansion duroplasty.^37^ One study attempted to enhance SCPP via intravenous fluids and/or vasopressor infusions to elevate MAP^45^ and another by vasopressor infusion alone.^34^

Adverse events were not reported in 20 original research articles and two studies stated that no adverse events occurred that were related to pressure monitoring. Among clinical studies monitoring ISP, adverse events include pseudomeningocele (n = 7 studies), CSF leak (n = 7), wound infection (n = 2), and syrinx (n = 1) – it should be noted that these adverse events were reported in studies of the same cohort of patients and therefore may be over-reported here. One clinical study that monitored ITP reported headache in a single patient and one study that monitored both ISP and ITP reported pseudomeningocele in 6/13 patients and CSF leak in 4/13, however it is unclear if this was related to the ISP or ITP monitoring.

### 3.4 Key themes

While there are a limited number of original research articles on the topic, and that even fewer have reported on attempts to influence SCPP, there are many literature reviews, perspectives, and editorials on the topic. Though not the focus of this review, our search identified 16 relevant original research articles that assessed functional recovery of which three are pre-clinical. The association between SCPP and neurological outcome has been systematically reviewed elsewhere by Thygesen et al. (2021).^13^ Thygesen et al. identified six studies (including one pre-clinical) and found that, despite growing support, it is not clear whether SCPP is related to neurological recovery due to the limited literature available. That this review identified more studies regarding recovery is likely explained by the stricter inclusion criteria of Thygesen and colleagues.

Another theme within the literature was the safety of ISP measured at the injury site and lumbar ITP monitoring. Regarding ISP, Phang et al. (2016) reported on the adverse events of 42 consecutive patients admitted to the neurointensive care unit at St. George's Hospital in London, United Kingdom.^38^ Eight of 42 patients experienced pseudomeningocele (19%), three had CSF leak (8%), and in one patient the probe became dislodged. Phang and colleagues concluded that monitoring from the injury site is safe and this was concurred by the systematic review of Menacho & Floyd (2021).^14^ More recent studies have reported the incidence of pseudomeningocele to be as high as 54% (7/13)^24^ and CSF leak in 19% of patients (5/26).^44^ Only two original research articles have addressed adverse events related to lumbar ITP monitoring. In a 2009 study by Kwon et al., one participant reported headache associated with the placement of a lumbar intrathecal catheter; this individual had a lumbar catheter inserted but CSF was not actively drained to reduce ITP.^31^ More recently, Yue and colleagues (2020) reported no adverse events in 15 patients undergoing ITP monitoring.^45^ Further discussion on the debate around the best method for measuring pressure within the intrathecal space, its relation to SCPP, spinal cord perfusion, and neurological recovery, is provided below.

## 4 Discussion

This scoping review aimed to catalog all research articles relevant to the significance of SCPP in acute traumatic SCI. The scoping review protocol allowed for more lenient inclusion criteria and as such we have collated the largest review on this topic, or related topics, to date. By synthesizing the literature we were able to identify key themes as well as research gaps and priorities for which we provide discussion below.

### 4.1 Can SCPP be enhanced by interventions that target increasing MAP?

Given recommendations from the American Academy of Neurologic Surgeons and Congress of Neurological Surgeons regarding the acute hemodynamic management of traumatic SCI,^3^^,^^4^ coupled with the relative ease with which to manage MAP in the neurointensive care unit, it is somewhat surprising that the literature search identified only two articles that explicitly attempted to increase SCPP by increasing MAP. Of course, assuming that ISP/ITP remains constant, increasing MAP will concurrently increase the calculated SCPP. However, there is a paucity of literature on the influence of vasopressor infusion on pressure within the intrathecal space.

Martirosyan et al. (2015) examined the efficacy of elevating MAP alone or in combination with CSF drainage to reduce ITP on SCPP and spinal cord blood flow in a porcine model of T5 SCI.^34^ In a subset of animals that underwent MAP elevation alone – via continuous phenylephrine infusion - there was a paradoxical reduction in SCPP due to a concomitant increase in ITP. However, when MAP elevation was combined with CSF drainage to lower ITP there was a 24% improvement in spinal cord blood flow but no mention of its influence on SCPP.

An increase in ITP was also noted with the administration of dopamine in a small series of human patients with SCI, as reported by Altaf et al. (2017)^41^ Notably, the administration of norepinephrine did not increase ITP.

In the clinical study of Yue et al. (2020), 15 patients underwent MAP augmentation via a combination of intravenous fluids and vasopressor infusion to maintain SCPP ≤65 mmHg.^45^ The purpose of this study was simply to report on the safety of a protocol targeting SCPP rather than its relation to functional recovery. The authors deemed SCPP target directed care to be feasible and without complications.

Interestingly, the systematic review of Tykocki and colleagues (2017) recommends maintaining a MAP of 110–130 mmHg to achieve an optimal SCPP of 90–100 mmHg given that ISP is typically 20–40 mmHg.^15^ Such a MAP recommendation is substantially higher than the aforementioned clinical guidelines and, regardless of feasibility, may put patients at greater risk of hemorrhage within the cord or cardiac events.^86^^,^^87^

### 4.2 Can SCPP be enhanced by interventions that target reducing pressure within the intrathecal space?

The influence of CSF drainage on pressure within the intrathecal space has been studied in two clinical studies to date. Neither study demonstrated a clear relationship between CSF drainage and pressure within the intrathecal space. Both studies implemented a conservative drainage protocol whereby a maximum of 10 mL CSF could be drained each hour – further, in the study by Hogg et al. (2020) a maximum of 30 mL could be drained in a 24-h period.^27^ Kwon et al. (2009) suggest a higher limit on drainage should be set in future studies of CSF drainage in SCI.^31^ Accordingly, in the setting of thoraco-abdominal aortic aneurysm repair, the drainage of up to 500 mL of CSF may be required to reduce CSF pressure to less than 10 mmHg.^12^ Guidelines for CSF drainage in the setting of traumatic brain injury suggest continuous rather than intermittent drainage to lower ITP and enhance recovery.^10^ Both clinical SCI studies attributed variability in results between patients to the patency of the intrathecal space. As mentioned above, in a porcine model of SCI, Martirosyan and colleagues (2015) found that CSF drainage combined with MAP augmentation was effective in enhancing spinal cord perfusion and was more effective than drainage alone.^34^

Maybe the most effective approach to reducing ITP via lumbar CSF drainage was that of Horn et al. (2008).^28^ In a rabbit model of SCI, CSF was drained via catheter inserted at the lumbosacral junction until ITP was equal to 10 mmHg regardless of the CSF volume drained. Histology indicated that drainage reduced the area of tissue damage at the injury site but this was not associated with functional recovery. One of the potential complications of draining CSF from the lumbar cistern and lowering the ITP is the risk of spinal cord herniation due to the generation of large pressure gradients. However, to date this complication has not been reported in the setting of acute traumatic SCI and, anecdotally, we are not aware of any reports of patients with acute traumatic SCI who have suffered such a complication.

Another method that has been trialed to reduce pressure within the intrathecal space is spinal cord cooling to prevent spinal cord swelling and reduce ITP. This method initially showed promise in a rodent model of SCI^28^ but a subsequent clinical trial was terminated early due to adverse events related to the cooling procedure rather than pressure monitoring.^51^

A more promising intervention to reduce ISP and enhance SCPP is that of expansion duroplasty during surgical decompression. To examine the relationship between duroplasty and SCPP, Phang et al. (2015) performed laminectomy and duroplasty in ten patients with traumatic SCI and found that, compared to laminectomy alone, the addition of duroplasty increased intradural space and more effectively decompressed the cord resulting in lower ISP and higher SCPP.^37^ Recent research suggests that expansion duroplasty may reduce the degree of the secondary injury and promote neurological recovery by minimizing edema and the subsequent obstruction of microcirculation, ischemia, and damage to neural tissue at the injury site.^88^, ^89^, ^90^

### 4.3 Where should pressure in the intrathecal space be measured?

The topic of where pressure in the intrathecal space should be measured – at the injury site or in the lumbar cistern caudal to the injury site - has generated some discussion (for example, see the letter to the editor and its response regarding the work of Martirosyan and colleagues^77^^,^^79^).

The insertion of a pressure sensor into the subdural space at the injury site can provide a measure of pressure within the injured cord when the cord swells and pushes the pressure sensor against the inelastic dura.^43^ Here, the ‘intraspinal pressure’ is likely to be different than the pressure measured in the lumbar cistern (i.e. ITP), as the occlusion of the subdural space by the swollen spinal cord creates a pressure gradient across the injury site.^91^ Hogg and colleagues (2020) demonstrated that when such swelling occurs and the subdural space is occluded ISP is typically ∼5 mmHg higher than ITP.^27^ However, in situations where such swelling and subdural occlusion is not present, there is a close correlation between ISP and ITP. This points to the importance of performing a multi-level posterior laminectomy/decompression at the time of surgical stabilization, as it has been shown in patients with severe cervical SCI that complete occlusion of the subdural space is infrequent if a wide laminectomy is performed.^92^

While measuring ISP at the injury site may provide a more accurate reflection of pressure within the cord, it does require the subdural insertion of a pressure sensor directly at the injury site. This has been associated with incidences of pseudomeningocele and CSF leakage and raises the potential for meningitis if the surgical wound were to become infected.^38^ Although these complications have not been associated with long-term consequences they may discourage implementation of this practice. Additionally, the approach conceptually requires that a “space occupying lesion” (the size of the calibre of the pressure probe) be inserted into the subdural space and to be compressed against the dura, which may also lessen the surgeon's enthusiasm for implementation.

In contrast, the insertion of a drainage catheter within the lumbar cistern is a common procedure and has a relatively low complication profile.^93^^,^^94^ However, it too can result in infectious meningitis or CSF leak, although these have been rarely reported. For example, Grady and colleagues found no neurologic deficits in over 500 patients undergoing neurosurgery in whom needles were placed in the subarachnoid space for the purpose CSF drainage.^95^ Inserting a needle and intrathecal catheter into the epidural space does engender a small risk of epidural hematoma. As such, the most recent clinical practice guidelines recommend the use of low molecular weight heparin to reduce the incidence of thromboembolic disease in SCI patients.^96^

The drainage of CSF to reduce ITP is also a standard technique. For example, in the setting of thoraco-abdominal aortic aneurysm repair - wherein the cords blood supply is vulnerable – ITP is monitored and CSF drained from the lumbar cistern.^12^ While this approach has significant advantages from a “simplicity” and “clinical familiarity” standpoint, it does suffer the potential for the ITP to not be representative of the ISP, especially in situations where the subdural space is occluded by the swollen spinal cord.^27^ In this regard, the success of such ITP monitoring is closely linked to the method by which the surgical decompression is performed, as achieving a patent subdural space around the cord is an important element of this type of monitoring.

### 4.4 Research gaps

This scoping review has highlighted a number of research gaps in the research and implementation of SCPP monitoring in acute traumatic SCI. For example, given that 23/24 original research studies in the clinical setting were conducted at only two research hospitals we wonder as to the feasibility of invasive monitoring at smaller centers. In that respect, the work of Hogg et al. (2021) that examined non-invasive predictors of optimum SCPP in lieu of invasive ISP monitoring is of particular interest.^26^

Second, it appears that little is known regarding CSF physiology following SCI beyond the fact that ITP is higher in the acute stage.^31^ Given the known influences of anaesthesia, pharmacological intervention, sleep disturbances, and posture on CSF physiology^97^^,^^98^ - how do these influence CSF volume, ITP, SCPP, and functional recovery in the setting of SCI? A better understanding of CSF physiology following SCI may accelerate interventions to reduce pressure around the injured cord.

Regarding interventions that target reducing pressure within the intrathecal space, Leonard and colleagues conducted a line of promising, novel research that highlighted potential pharmacological targets for reducing neurogenic inflammation (substance P) and reducing CSF secretion and/or accumulation (by targeting aquaporin water channels) to reduce ITP.^32^^,^^33^ Our literature search did not identify any more recent studies on such interventions as they relate to SCPP in SCI and there remains scope for studies of pharmacological intervention to reduce ITP in the setting of SCI.

### 4.5 Strengths and limitations of the scoping review

The broad research question adopted in this scoping review may be considered both a strength and limitation. For one, it allowed us to map all literature on the significance of SCPP in SCI including articles that previous reviews have overlooked. However, a broad research question may result in grey areas in regard to whether certain articles should or should not be included in the review. As we explicitly searched for papers that discussed, or were relevant to, SCPP monitoring we may have overlooked studies of ISP/ITP that were not relevant to SCPP.

That the majority of original research is from two group raises the question of duplicate or re-analysis of data. For example, Squair et al. (2017), Squair et al. (2019), and Gee et al. (2022) indicate that they report on patients from the same dataset. However, this is not always disclosed which may lead to challenges if/when a meta-analysis is undertaken to calculate the effect of SCPP monitoring in acute traumatic SCI.

## 5 Conclusions

This scoping review systematically synthesized articles related to the significance of SCPP monitoring in acute traumatic SCI. Despite a growing body of literature on the importance of SCPP many questions remain unresolved. We have highlighted a number of research gaps in the literature that we believe, if addressed, may provide insight into the most effective way to monitor SCPP, interventions to alter SCPP, and whether SCPP determines functional recovery following traumatic SCI.

## Funding

BKK holds a Canada Research Chair in Spinal Cord Injury and Dvorak Chair in Spine Trauma.

## Declaration of competing interest

The authors declare no conflict of interest.
